# Using modified trapping regimes to understand the behavioral and spatial ecology of *Philornis downsi* (Diptera: Muscidae)

**DOI:** 10.1093/ee/nvae014

**Published:** 2024-03-14

**Authors:** Rebecca A Boulton, Andrea Cahuana, Paola F Lahuatte, Erika Ramírez, Christian Sevilla, Charlotte E Causton

**Affiliations:** Biological and Environmental Sciences, Faculty of Natural Sciences, University of Stirling, FK9 4NF, UK; Laboratory of Genetics, Plant Sciences Group, Wageningen University and Research, 6708 PB, The Netherlands; Charles Darwin Research Station, Charles Darwin Foundation, Santa Cruz, Galapagos Islands, Ecuador; Charles Darwin Research Station, Charles Darwin Foundation, Santa Cruz, Galapagos Islands, Ecuador; Charles Darwin Research Station, Charles Darwin Foundation, Santa Cruz, Galapagos Islands, Ecuador; Galapagos National Park Directorate, Santa Cruz, Galapagos Islands, Ecuador; Charles Darwin Research Station, Charles Darwin Foundation, Santa Cruz, Galapagos Islands, Ecuador

**Keywords:** Muscidae, Diptera, invasive species, trapping, ectoparasite

## Abstract

The avian vampire fly *Philornis downsi* (Dodge & Aitken) (Diptera: Muscidae) is native to continental South America and the Caribbean, but invasive in the Galapagos Archipelago. The larvae of *P. downsi* feed on the blood and tissues of the nestlings of 75% of the small land bird species that are endemic or native to Galapagos, causing high in-nest mortality and severe population declines in some species. Efficient trapping techniques are vital to safeguarding these birds in the short term as well as for monitoring fly populations, but basic information about the ecology of the fly is still needed to help develop a species-appropriate trapping method. In this study, we used a novel trapping regime with a vertical distribution to make inferences about *P. downsi*’s behavioral and spatial ecology and to optimize trap catch. Our results showed that male and female *P. downsi* were trapped in greater numbers below the canopy (3.1–7.5 m), lower down than other commonly caught insect species (5.1–11.5 m). Notably, the effect of trap height remained consistent across seasons and different weather conditions. These findings suggest that *P. downsi* tend to move at heights where their hosts nest (at or below the canopy) and do not spend time above the canopy. This also makes it unlikely that strategies such as hill-topping or aerial swarming are being used to locate mates. As such, trapping and control efforts should be focused below the canopy in forests with similar canopy heights to effectively capture *P. downsi* and reduce bycatch of other insects.

## Resumen

La mosca vampiro aviar *Philornis downsi* (Dodge & Aitken) (Diptera: Muscidae) es originaria de América del Sur continental y el Caribe, pero invasora en el archipiélago de Galápagos. Las larvas de *P. downsi* se alimentan de la sangre y los tejidos de los polluelos de 75% de las aves terrestres pequeñas que son endémicas o nativas de Galápagos, lo que provoca una alta mortalidad en los nidos y graves disminuciones en las poblaciones de algunas especies. Las técnicas de captura eficientes son vitales para salvaguardar a estas aves a corto plazo, así como para monitorear las poblaciones de moscas, pero aún se necesita información básica sobre la ecología de *P. downsi* para ayudar a desarrollar un método de captura apropiado para esta especie. En este estudio utilizamos un nuevo régimen de captura con una distribución vertical para hacer inferencias sobre la ecología espacial y conductual de *P. downsi* y optimizar la captura con trampas. Nuestros resultados mostraron que los machos y las hembras de *P. downsi* quedaron atrapados en mayor número debajo del dosel (3,1–7,5 m), más abajo que otras especies de insectos comúnmente capturadas (5,1–11,5 m). En particular, el efecto de la altura de la trampa se mantuvo constante en todas las estaciones y en diferentes condiciones climáticas. Estos hallazgos sugieren que *P. downsi* tiende a moverse en las alturas donde anidan sus huéspedes (en o debajo del dosel) y no pasa tiempo por encima del dosel. Esto también hace que sea poco probable que se utilicen estrategias como hill topping o el enjambre aéreo para localizar parejas. Como tal, los esfuerzos de captura y control deben centrarse debajo del dosel en bosques con alturas de dosel similares para capturar eficazmente *P. downsi* y reducir la captura incidental de otros insectos.

## Introduction

Diverse methods have been developed to trap and collect animals for biodiversity surveys, but the choice of methods used can have far-reaching consequences for interpreting ecology and behavior (i.e., Hayes 1970, Nichols et al. 1984, Epsky et al. 1999, 2014, Michelangeli et al. 2016). Developing species-appropriate trapping methodologies is not only critical for the accurate assessment of species abundance and diversity but can also contribute valuable additional data regarding habitat use, behavioral ecology, and even sensory capacity. This information is not only informative for ecologists but can be used to design more effective, species-specific traps to monitor or control pests and invasive species (Epsky et al. 2014). For instance, leafhoppers (Hemiptera: Cicadellidae) are economically important plant pests that live alongside natural enemies (predators and parasitoids) that can help to control them. Rodriguez-Saona et al. (2012) investigated how trap height and color can be manipulated to maximize leafhopper capture rates but reduce bycatch of beneficial insect species. Designing a species-appropriate trapping regime is an important first step when beginning to characterize the population dynamics and ecology of little-known species, or species that are new to an area. Such work sets the foundation for more detailed follow-up work and, where necessary, control and management (Byers 2012).

In this study, we apply these approaches to the avian vampire fly, *Philornis downsi* (Diptera: Muscidae) (Dodge & Aitken). This species is native to continental South America but invasive in Galapagos, where it attacks at least 75% of the small native and endemic land bird species and is considered the leading cause of decline in several species that are threatened with extinction (Fessl et al. 2006, 2018, Kleindorfer et al. 2019). Adult *P. downsi* are free-living but adult females oviposit in bird nests. The fly larvae then hatch and feed on the blood of developing chicks, causing high mortality across bird species (Fessl et al. 2018). Since the ramifications of this invasion have been realized, greater attempts have been made to study the population dynamics, ecology, and natural history of this species in its native and introduced range with the hope that these insights can inform more effective control (Bulgarella et al. 2015, 2022, Kleindorfer and Dudaniec 2016, Kleindorfer et al. 2016, Knutie et al. 2016, Causton et al. 2019, Cimadom et al. 2019, Common et al. 2021, Pike et al. 2021). While much progress has been made in understanding *P. downsi*, there are still substantial gaps in our knowledge of this species basic ecology and reproductive behavior (Fessl et al. 2018). Furthermore, current trapping techniques for control or monitoring purposes have been shown to be inefficient in capturing flies (Causton et al. 2019), requiring the development of a trapping regime that is specific to *P. downsi*.

A 2.5-yr study of the population dynamics of *P. downsi* on Santa Cruz Island (Causton et al. 2019) found that adult *P. downsi* were more abundant in traps in the hot season (usually between January and May) when more resources (bird hosts) are available for reproduction (Lack 1950). This pattern is similar to many other arthropod species in Galapagos that rely on the resources that become available after the strong rains associated with the hot season (i.e., Schluter 1984, Peck 2001, Roque-Albelo 2006), increasing the potential for bycatch of non-target species. With this in mind, in this study, we set out to test whether traps deployed at different heights in the canopy caught different numbers of *P. downsi*, as well as other insects, and whether these spatial patterns vary seasonally with changes in the weather. The data collected were also used to learn about other aspects of *P. downsi*’s behavior. For instance, if flies were caught at different heights in the bird breeding and non-breeding season it might hint at the types of resources the adults feed on when they are not searching for hosts. Furthermore, sex-specific patterns of trapping could be used to make inferences about its mating system; if male *P. downsi* are found above the canopy this might indicate that hill-topping (where males aggregate at higher elevations and receptive females move upwards to mate) occurs in this species (Wilkinson & Johns 2005, Kokko et al. 2014). The results of this study provide key information about the ecology of *P. downsi* and will also assist with international efforts (e.g., Cha et al. 2016, Mieles 2018) to develop novel mass trapping methods for reducing *P. downsi* populations in the nesting areas of at-risk bird species unique to this archipelago.

## Materials and Methods

We used a trapping regime that tests whether trap height influences capture rates of male and female *P. downsi* to answer the following questions: (i) Do trap rates of *P. downsi* vary with trap height and is this pattern consistent across male and female flies? (ii) Is height-dependent variation in trap rate consistent over the bird breeding and non-breeding seasons? (iii) Are the spatial and temporal trapping patterns of *P. downsi* similar to other insect species with overlapping niches/ranges?

### Study Site

This study was carried out in the highland region at Los Gemelos (0° 37’82.0”S, 90°23’44.4”W, elevation 589–616 m), Santa Cruz Island, Galapagos, Ecuador between March 2016 and May 2018. This site is vegetated primarily by endemic *Scalesia pedunculata* trees with much of the understory covered in invasive blackberry, *Rubus niveus*, but also invasive *Cestrum auriculatum* and *Tradescantia fluminensis* (Rentería et al. 2012, Jäger et al. 2017). This highland region typically experiences sporadic rain showers during the hot season (January–May) and an extended moister period during the cooler season (June–December) characterized by low-hanging clouds (Trueman and d’Ozouville 2010, Walentowitz et al. 2021).

### Trapping Procedure

#### Trap installation and height.

**Fig. 1.**
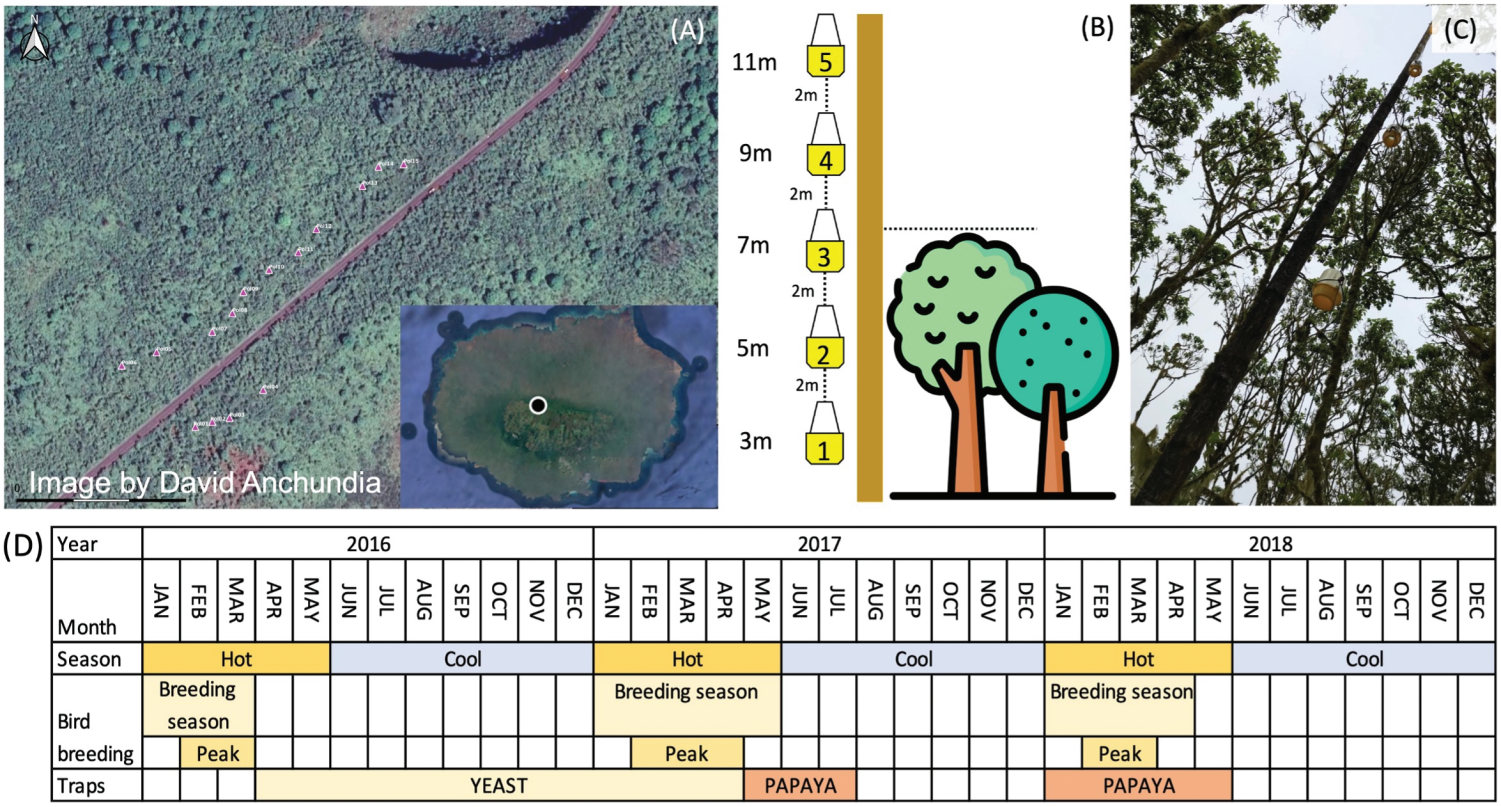
a) Los Gemelos forest where 15 poles were placed (indicated by triangle markers), insert shows location of Los Gemelos on Santa Cruz Island (image by David Anchundia). b) Schematic showing trapping design with 5 trap height categories relative to the top of the canopy (tree icon made by Freepik from www.flaticon.com). c) Trapping pole after installation in Los Gemelos. d) Trap bait type deployed over the course of the study shown alongside season (hot/cool) and bird breeding status.

A total of 15 bamboo poles approximately 14 m high and 20 cm diameter were installed in the Los Gemelos forest (Fig. 1a). The poles were placed near *S. pedunculata* trees in semiopen areas with 10–15 m between each pole. Each pole had 5 yellow McPhail traps (Naturquim, Ecuador) attached vertically, positioned at regular spaced intervals. Traps were hung on a cord with 2 m between each trap (from the base of 1 trap to the next) and the cord connected to a pulley system at the top of the pole (Fig. 1b and c). As a reference point, the top of the canopy was aligned with the third trap (trap 3) on each pole (*x̄* = 6.22m ± 0.59 SD). This was to ensure that the traps on each pole were always hung at the same level with respect to the canopy. The traps were found in the following height range: trap 1 (3.1–5.5 m), trap 2 (5.1–7.5 m), trap 3 (7.1–9.5 m), trap 4 (9.1–11.5 m), and trap 5 (11.1–13 m).

#### Data collection.

Traps were set out for 48 h every 2 wk from 30 March 2016 to 27 July 2017 and from 16 Jan 2018 to 24 May 2018 giving a total of 48 sampling events (total *N* = 3,255 traps). The trapping periods covered the end of the bird-breeding season and the full non-bird breeding season in 2016 and 2 complete bird-breeding seasons in 2017 and 2018 (Fig. 1d). Two days after deployment, traps were lowered and the number of male and female *P. downsi* counted. We also counted other insects commonly found in traps to provide an additional comparison to trap rates of *P. downsi*. We did not attempt to identify these specimens to the species level as this was beyond the scope of this study, instead, we categorized other insects found in traps as Diptera (other than *P. downsi*), moths (Lepidoptera), and the invasive wasp *Polistes versicolor*.

Traps set out between 30 March 2016 and 5 May 2017 were filled with 125 ml of a yeast and sugar solution, which had shown to be attractive to *P. downsi* (Cha et al. 2016). Traps set out after this date (and until the end of the experiment) were filled with 125 ml of a 3-day fermented papaya and sugar mix (Causton et al. 2019) as preliminary data suggested that papaya traps might be more effective than yeast at trapping *P. downsi.* Note, that because of this the experimental design was not paired as bait type was changed during the study at the same time for all traps (see Fig. 1d for an overview of when traps were set out and with which bait type).

#### Weather data.

We used a dataset that combined records across weather stations in the highlands of Santa Cruz Island (Walentowitz et al. 2021) to estimate the average daily rainfall (mm), temperature (°C), and % humidity over each 48-h trapping period at Los Gemelos. The dataset combined weather data at the trapping site at Los Gemelos (when available) and measurements from El Carmen (Rolf Sievers farm; 0˚39’57.49”S, 90˚22’35.04” W; ~200 m below the study site), Santa Rosa (0°39’00.9”S 90°24’26.5”W; ~200 m below the study site), and Bellavista (0°41’32.6”S 90°19’41.4”W; ~400 m below the study site).

#### Data analysis.

Generalized linear mixed models (using the R package *glmmTMB;*Brooks et al. 2017) with a negative binomial error structure were used to test whether *P. downsi* trap rates (the number of flies caught over each 48-h period) varied over different heights and climates. Models included trap height as a fixed effect with 5 levels (trap 1: 3.1–5.5 m, trap 2: 5.1–7.5 m, trap 3: 7.1–9.5 m, trap 4: 9.1–11.5 m, and trap 5: 11.1–13 m). To control for the confounding effect of bait type (which was changed during the experiment), bait type (2 categories: papaya and yeast) was included as a fixed effect in all models. Trap ID (per trap, not per pole) was included as a random effect to account for non-independence of repeated counts from the same traps. We also included climatic variables in the model (rainfall (mm), temperature (°C), and % humidity) as continuous covariates after checking for multicollinearity (using the function *omcdiag* in the package *mctest* in Rstudio; Ullah et al. 2016). Interaction effects between trap height and all climatic variables were fitted. We also included study week as a covariate to investigate seasonal changes in trap rates.

To test whether the effects of height and weather were the same for male and female *P. downsi*, a binomial GLMM (in the package *lme4*; Bates et al. 2015) was used containing the same predictors and random effects but the response variable was the count of male and female *P. downsi* (modeled using the *cbind* function; note that interaction effects between weather variables and trap height were not fitted for this model due to non-convergence indicating a lack of fit).

To test the effect of trap height and weather on species other than *P. downsi*, we fitted the same predictors (trap height, bait type, rainfall, temperature, humidity, and study week) and random effects (trap ID) in a GLMM (using *glmmTMB*) but the response variables were counts of either Diptera, moths, or *P. versicolor* and the error structure was negative binomial.

Generalized additive mixed models (GAMM; using the R package *mgcv*; Wood 2017) with a negative binomial error structure were used to test whether height effects of trapping were consistent across the bird breeding and non-breeding seasons in *P. downsi*. The response variable was the total count of *P. downsi* per trap and trap ID was included as a random effect. We ran 2 models, one where a Duchon spline was fitted to week of year and another which fitted separate Duchon splines for each of the 5 different height ranges. We compared the model fit using the Akaike information criterion (AIC) to determine whether seasonal variation in *P. downsi* trap rate differs based on trap height. We used the same GAMM structure but with the 3 other species groups collected (Diptera, moths, and *P. versicolor)* as the response variables to test whether patterns of annual variation in trap rates of other insect species mirrored those observed in *P. downsi*. For these models, the error structure was Poisson (with an observation level random effect to account for overdispersion; Harrison 2014) for moths and Diptera and negative binomial for *P. versicolor.* We ran an additional negative binomial GAMM to test for sex-specific differences in *P. downsi* trap rate over the year. We tested whether fitting separate week splines for male and female *P. downsi* improved GAMM fit based on the AIC.

All analyses were conducted in R (R Studio team 2016). We tested whether all models were appropriately specified using the packages DHARMa (Hartig 2020) and gam.check in *mgcv* (Wood 2017).

## Results

### Trap Rates of *P. downsi*

**Fig. 2.**
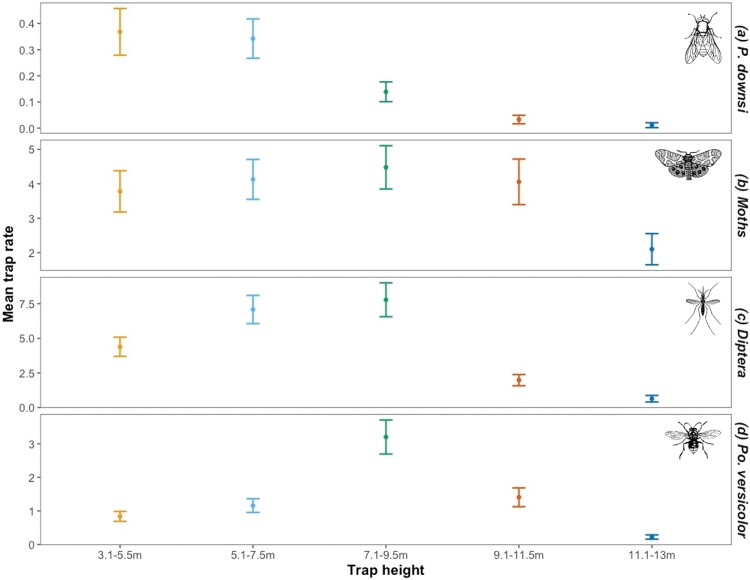
Effect of trap height on mean trap rates of a) *P. downsi*, b) moths, c) other Diptera, and d) *Polistes versicolor* (error bars are 95% CIs) across the entire study period.

Across the 48 trapping events we caught a total of 622 *P. downsi.* We found that there was a strong negative effect of increasing height on *P. downsi* trap rates, with the highest traps catching the fewest flies (Table 1; Fig. 2a). As bait type was changed during the experiment, we included it in the models; while there was a significant effect of bait in the models this did not influence the effect of trap height, which was consistent across both bait types (Table 1).

**Table 1.** Results of generalized linear mixed models for trap rates of *P. downsi*, moths, Diptera (excluding *P. downsi*), and *P. versicolor* (significant effects in bold)

Species	Variable	*X* ^2^	*df*	*P*
*P. downsi*	**Trap height**	**87.62**	**4**	**<0.001**
	**Bait type**	**177.82**	**1**	**<0.001**
	**Temperature**	**169.37**	1	**<0.001**
	**Rainfall**	**170.41**	**1**	**<0.001**
	Humidity	2.93	1	0.09
	**Study week**	**71.38**	**1**	**<0.001**
	Trap height*Bait type	4.44	4	0.35
	Trap height* Temperature	2.67	4	0.61
	Trap height*Rainfall	1.75	4	0.78
	Trap height*Humidity	**37.92**	**4**	**<0.001**
Moths	**Trap height**	**86.78**	**4**	**<0.001**
	**Bait type**	**65.92**	**1**	**<0.001**
	**Temperature**	**172.98**	**1**	**<0.001**
	**Rainfall**	**43.67**	**1**	**<0.001**
	**Humidity**	**85.11**	**1**	**<0.001**
	**Study week**	**168.46**	**1**	**<0.001**
	Trap height*Bait type	4.52	4	0.34
	Trap height* Temperature	8.06	4	0.09
	Trap height*Rainfall	8.58	4	0.07
	Trap height*Humidity	**17.41**	**4**	**<0.001**
Diptera	**Trap height**	**342.25**	**4**	**<0.001**
	**Bait type**	**22.83**	**1**	**<0.001**
	**Temperature**	**80.15**	**1**	**<0.001**
	**Rainfall**	**24.25**	**1**	**<0.001**
	**Humidity**	**36.31**	**1**	**<0.001**
	**Study week**	**30.65**	**1**	**<0.001**
	Trap height*Bait type	**9.87**	**4**	**0.04**
	Trap height* Temperature	**19.49**	**4**	**<0.001**
	Trap height*Rainfall	1.29	4	0.86
	Trap height*Humidity	0.76	4	0.94
*P. versicolor*	**Trap height**	**147.11**	**4**	**<0.001**
	**Bait type**	**62.97**	**1**	**<0.001**
	**Temperature**	**460.20**	**1**	**<0.001**
	Rainfall	2.27	1	0.13
	**Humidity**	**19.54**	**1**	**<0.001**
	**Study week**	**251.42**	**1**	**<0.001**
	Trap height*Bait type	**30.49**	**4**	**<0.001**
	Trap height* Temperature	**22.88**	**4**	**<0.001**
	Trap height*Rainfall	2.34	4	0.67
	Trap height*Humidity	4.64	4	0.33

**Fig. 3.**
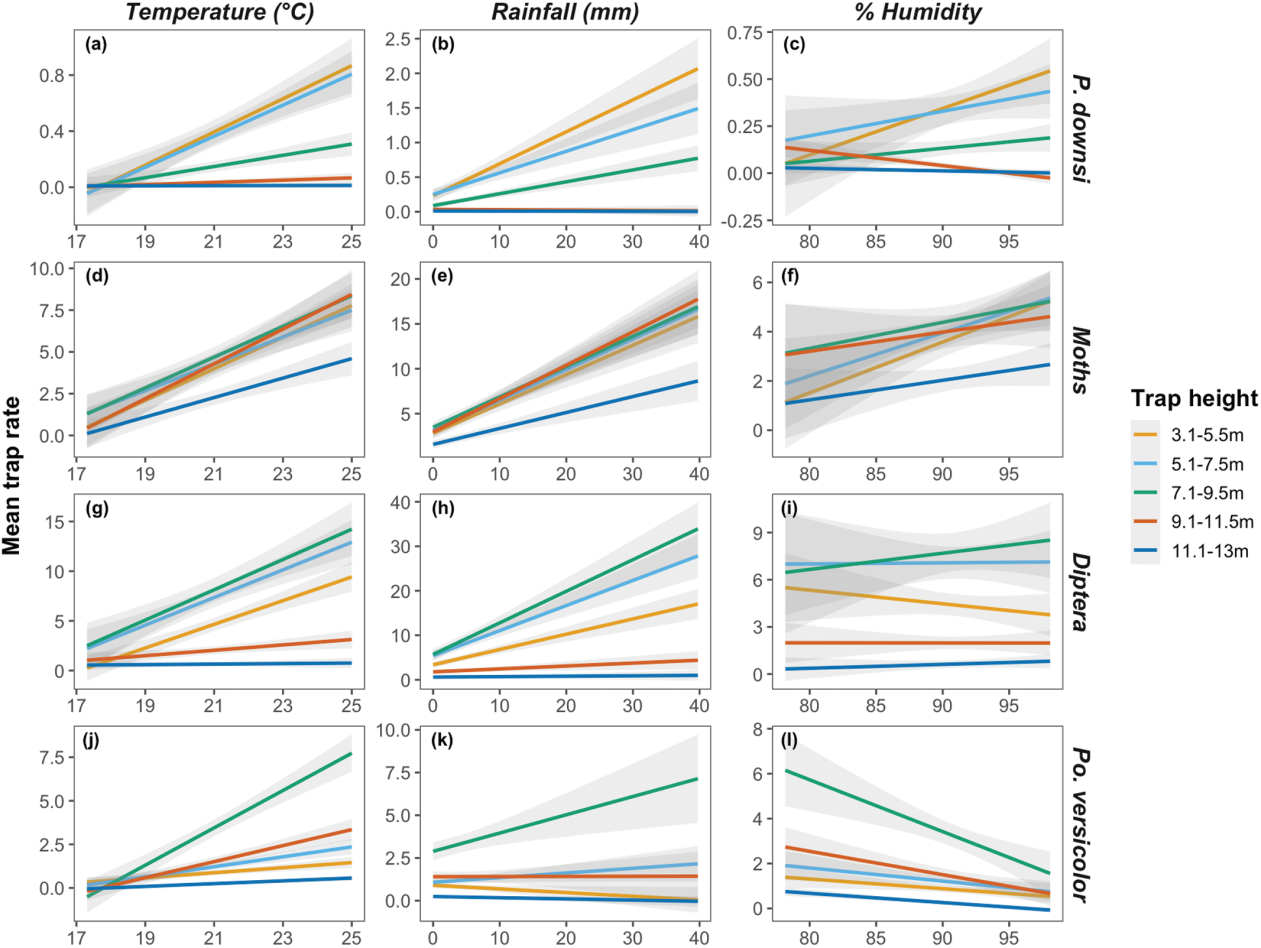
Effect of temperature (°C), rainfall (mm), and % humidity on trap rates of a)–c) *P. downsi*, d)–f) moths, g)–i) other Diptera, and j)–l) *P. versicolor*. Shaded areas are 95%.

Trap rates of *P. downsi* increased with temperature and rainfall (Table 1; Fig. 3a and b) but there was no effect of humidity on the trap rate (Table 1, Fig. 3c). There was however a significant interaction effect between humidity and trap height on the number of *P. downsi* caught (Table 1); trap rates increased with greater humidity but only in the 3 lowest traps (Table 1, Fig. 3c). Similar patterns were seen across all climatic variables with the most pronounced effects on trap rate occurring in the 3 lowest traps, but the interaction effects were not significant for rainfall or temperature (Table 1).

### Other Insects Trapped

In addition to *P. downsi*, traps caught 31,053 other insects which, with the exception of the invasive wasp *P. versicolor*, were identified to order. In total 12,362 Lepidoptera (almost entirely moths) 14,196 Diptera (other than *P. downsi*), and 4,495 *P. versicolor* were collected. As with *P. downsi,* the highest traps (at 11.1–13 m) caught few insects (Fig. 2b–d). For moths, trap rates were similar across all trap heights below 11.1–13 m (Fig. 2b). For other Diptera, trap rates were greatest in traps between 5.1 and 9.5 m (Table 1, Fig. 2c). For *P. versicolor* there was a pronounced peak in trap rates between 7.1 and 9.5 m (Fig. 2d).

For moths, other Diptera, and *P. versicolor*, the effects of temperature, humidity, and rainfall were consistent and showed the same general patterns as in *P. downsi* (Table 1; Fig. 3d-l). Trap rates increased with increasing temperature (Fig. 3d, g, and j), rainfall (Fig. 3e, h, and k), and humidity (Fig. 3f and i). The exception was that with *P. versicolor* humidity had a negative effect on the trap rate (Table 1; Fig. 3l). As was the case with *P. downsi*, the trends seen were generally only discernable in the traps at heights which caught reasonable numbers of insects. Any significant interaction effects that were detected appear to arise because of this artifact of sample size; there were no clear biologically significant differences in how weather influences trapping at different heights (see Table 1).

### Trap Rates of Male and Female *P. downsi* According to Trap Height and Weather

**Fig. 4.**
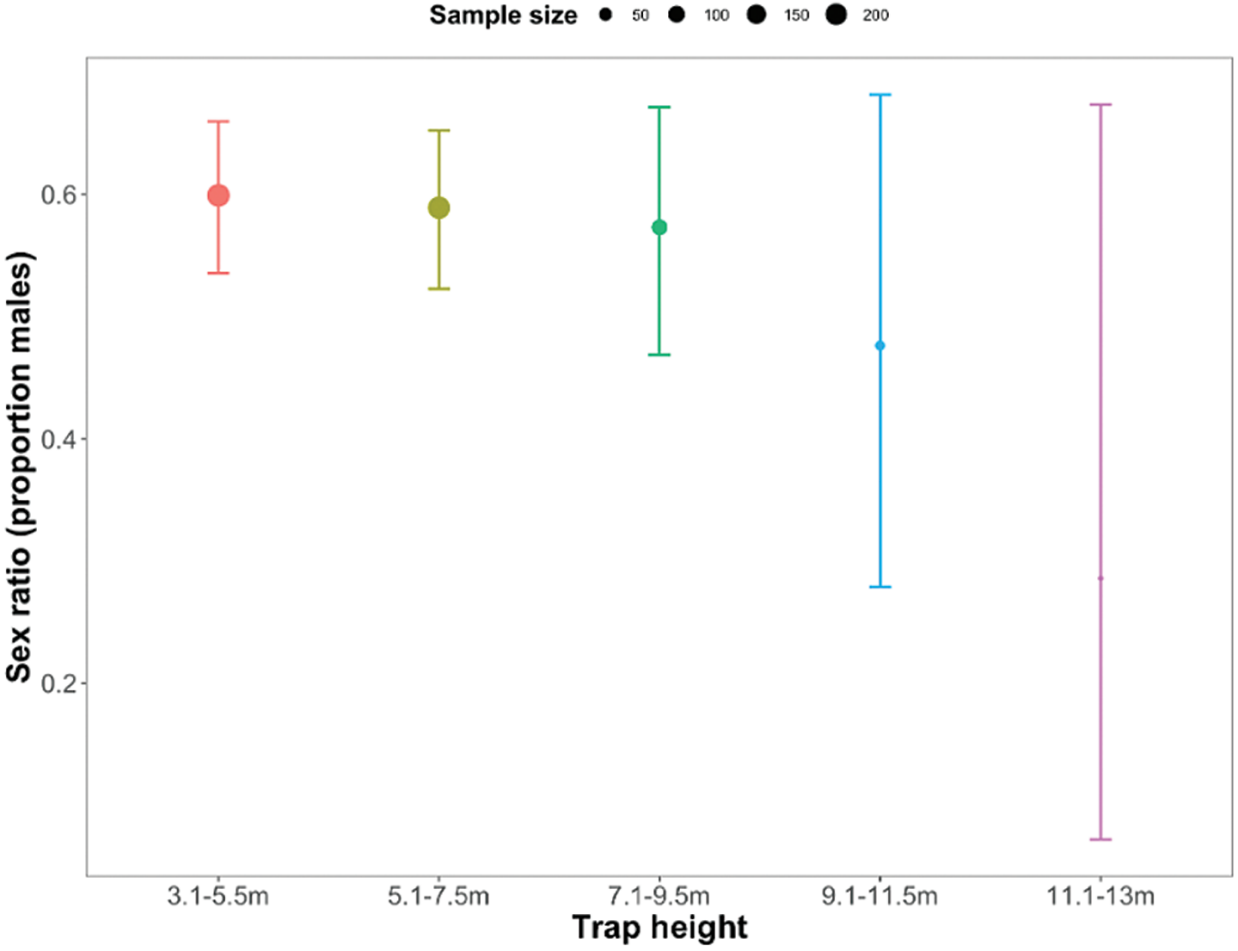
Effect of trap height on the sex ratio (proportion males) of *P. downsi* caught (error bars are binomial 95% confidence intervals).

On average the *P. downsi* sex ratio (proportion males) was 0.54 (±0.18 95% CI). The sex ratio of *P. downsi* caught was consistent across trap heights (*X*^2^ =1.96, *df* = 4, *P* = 0.74; Fig. 4).

**Fig. 5.**
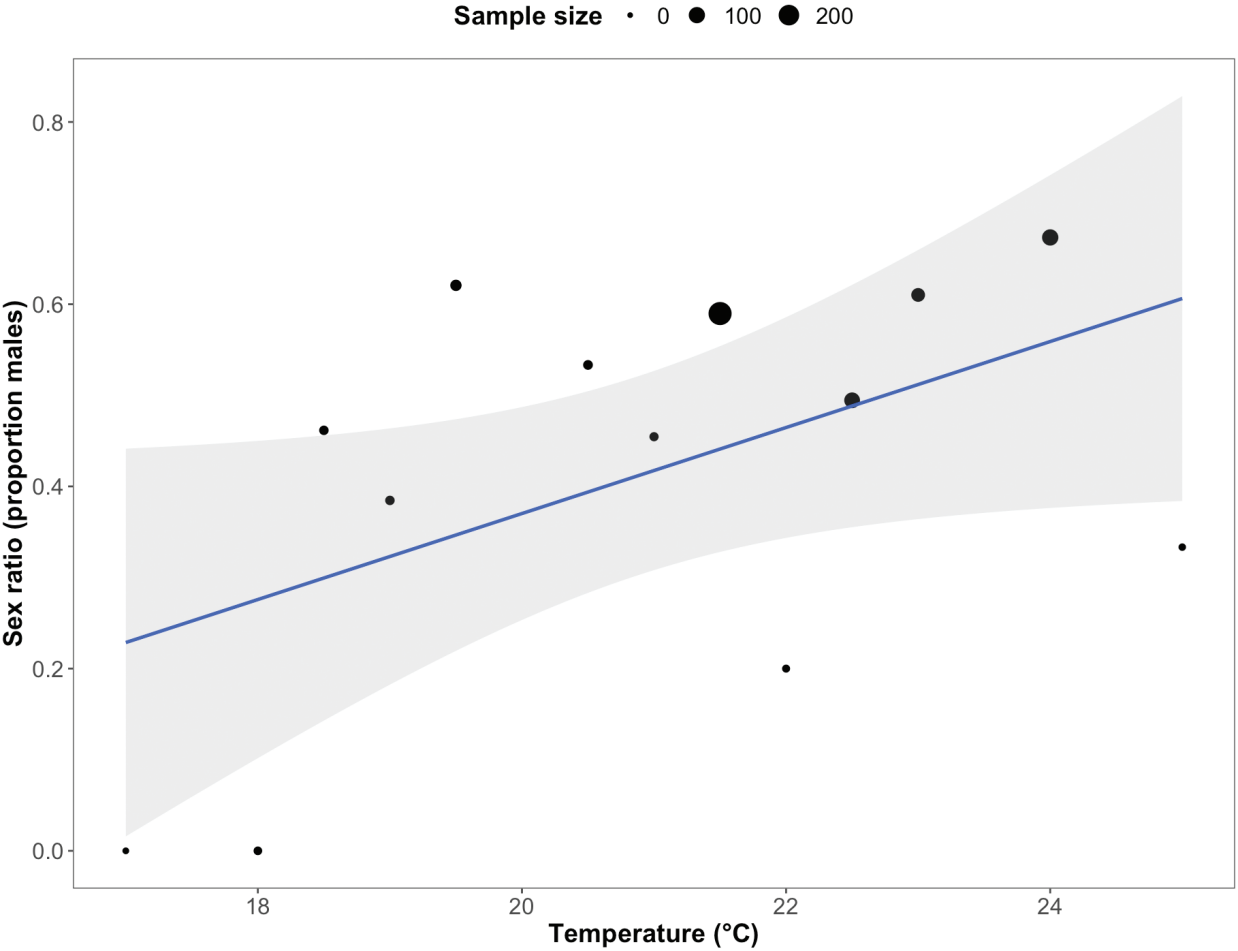
Effect of temperature on the proportion of male (sex ratio) *P. downsi* caught in traps. Shaded areas show 95% CIs and the size of the points is proportional to sample size (larger total numbers of *P. downsi* trapped are indicated by larger points).

There were no effects of humidity (*X*^2^ = 3.01, *df* = 1, *P* = 0.108) or rainfall on the sex ratio of *P. downsi* caught (*X*^2^ = 0.09, *df* = 1, *P* = 0.76). Temperature was the only climatic variable that was significantly associated with the sex ratio; with increasing temperatures proportionally more male *P. downsi* were trapped (*X*^2^ = 6.98, *df* = 1, *P* = 0.03; Fig. 5).

### Seasonal Variation in Height-Dependent Trap Rate for *P. downsi* and Other Species Groups

There were seasonal fluctuations in *P. downsi* trap rates across all trap heights, with a peak between weeks 15 and 20 (April–May). Fitting separate seasonal (week) splines for traps across the 5 height categories resulted in a poorer GAMM fit (AIC week only: 19,514.41, AIC week*trap height: 21,651.92). We did not see significant sex-specific variation in *P. downsi* trap rates across the season; fitting separate week splines for male and female *P. downsi* resulted in a poorer model fit (AIC week only: 42,918.22, AIC week*sex: 43,473.92, females: *F* = 22.64, *edf* = 10.27, *P* < 0.0001; males: *F* = 37.45, *edf* = 8.15, *P* < 0.0001).

As was the case for *P. downsi,* fitting separate splines for each trap height did not improve model fit for trap rates of other Diptera (AIC week only: 12,979.85, AIC week*height: 13,003.84) or moths (AIC week only: 13,003.70, AIC week*height: 13,133.46). For *P. versicolor*, fitting separate splines for each trap height did result in a lower AIC suggesting an improved model fit (AIC week only: 15,891.81, AIC week*height: 15,458.83).

**Fig. 6.**
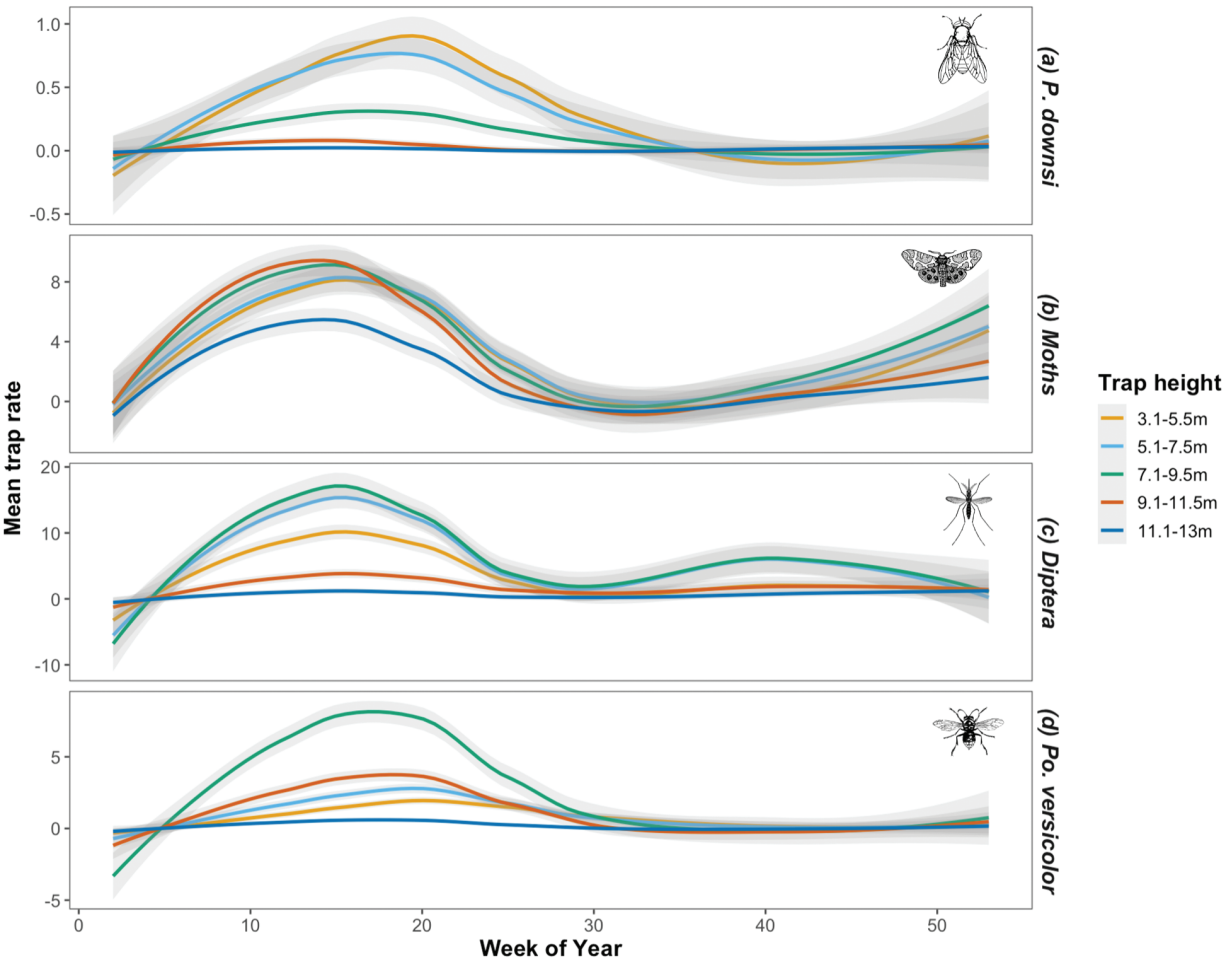
Fluctuation in numbers of a) *P. downsi*, b) moths, c) other Diptera, and d) *P. versicolor* captured every 2 weeks over the year at all 5 different trap height categories (Shaded areas are CIs estimated using the LOESS function).

Seasonal fluctuations in trap rates of other insects caught in traps aligned with those seen in *P. downsi,* with most peaking between April and May (weeks 15 and 20). In other Diptera, a second, smaller peak was also seen around week 40. This second peak was not observed in any of the other taxa monitored. For *P. downsi,* trap rates were consistently low in the 2 highest traps (Fig. 6a), resulting in low seasonal variation indicated by small, non-significant estimated degrees of freedom for the smoothing terms at these heights (Table 2). A similar pattern was seen in other Diptera, with the highest 2 traps catching the fewest individuals and showing the lowest variation in trap rates across the season (Fig. 6c). In *P. downsi* the lowest 2 traps (3.1–5.5 m and 5.1–7.5 m) showed the strongest patterns of seasonal variation and the highest peaks in trap rates between weeks 15 and 20 (April–May) (Fig. 6a). Whereas traps at moderate heights showed the most seasonal variation, and the most pronounced peaks between weeks 15 and 20 in trap rates of Diptera (5.1–9.5 m) and *P. versicolor* (7.1–9.5 m; Table 2; Fig. 6c and d). Only in the moths was seasonal variation in trap rates consistent across trapping heights (Fig. 6b; Table 2).

**Table 2.** Analysis of variance from generalized additive mixed models for trap rates across all species groups collected over the year across all heights

Species	Height	Mean trapped ± CI	*F*	Estimated *df*	*P*
*P. downsi*	11.1–13 m	0.02 ± 0.01	0.00	0.00	0.82
	9.1–11.5 m	0.04 ± 0.02	0.03	0.245	0.26
	7.1–9.5 m	0.15 ± 0.04	11.98	3.84	**<0.001**
	5.1–7.5 m	0.37 ± 0.07	29.08	6.59	**<0.001**
	3.1–5.5 m	0.38 ± 0.09	32.25	7.03	**<0.001**
Diptera	11.1–13 m	0.64 ± 0.23	10.48	9.82	**<0.001**
	9.1–11.5 m	1.99 ± 0.39	11.41	8.79	**<0.001**
	7.1–9.5 m	7.71 ± 1.21	28.02	9.07	**<0.001**
	5.1–7.5 m	7.01 ± 1.00	32.78	9.86	**<0.001**
	3.1–5.5 m	4.35 ± 0.68	25.84	9.42	**<0.001**
*P. versicolor*	11.1–13 m	0.24 ± 0.07	15.52	7.01	**<0.001**
	9.1–11. 5m	1.45 ± 0.28	36.21	8.83	**<0.001**
	7.1–9.5 m	3.17 ± 0.49	42.05	9.56	**<0.001**
	5.1–7.5 m	1.16 ± 0.21	25.05	9.93	**<0.001**
	3.1–5.5 m	0.86 ± 0.14	22.46	8.13	**<0.001**
Moths	11.1–13 m	2.24 ± 0.45	22.06	9.00	**<0.001**
	9.1–11.5 m	4.22 ± 0.66	30.34	8.98	**<0.001**
	7.1–9.5 m	4.53 ± 0.62	24.93	8.61	**<0.001**
	5.1–7.5 m	4.15 ± 0.57	22.12	9.23	**<0.001**
	3.1–5.5 m	3.80 ± 0.59	20.92	8.80	**<0.001**

## Discussion

In this study, we investigated spatial variation in trap rates of the invasive avian vampire fly, *P. downsi,* in the endemic *S. pedunculata* forest on Santa Cruz Island in the Galapagos archipelago. We found that trap rates of *P. downsi* were influenced by trap height, with greater numbers caught in traps at or below the canopy (3.1–7.5 m) than above the canopy (7.5–13 m). We observed pronounced seasonal and climatic fluctuations in *P. downsi*, with trap rates peaking in the hot season between April and May. Trap rates of *P. downsi* increased with increasing temperature (in particular for males), rainfall, and relative humidity, coinciding with the breeding season of their avian hosts (typically January–April/May; Lack 1950, Grant 1986, Causton et al. 2019). These patterns were more visible in the lower traps; in traps approximately 2 m or more above the canopy too few flies were caught to discern any seasonal or climatic variation. Notably, other species collected in the traps showed similar patterns to *P. downsi* according to climate and time of year. With the exception of moths, other species were, like *P. downsi*, caught in low numbers in the highest traps (11.1–13 m), but in contrast to *P. downsi*, all other species counted (*P. versicolor*, other Diptera and moths) were caught at the highest numbers in the 3 intermediate height traps (5.1–11.5 m).

The flight layer describes the vertical layer where an insect flies in search of food, mates, and oviposition resources (Byers 2012). In this study, we saw that the flight layer occupied by *P. downsi* was low down in the vertical strata of the forest (3.1–7.5 m), at or below the canopy of the dominant *S. pedunculata* vegetation, which is usually found at 7–8 m (Riegl et al. 2023). This flight layer could be related to the vertical distribution of its hosts. The nests of many passerine species are found within this height range (see Grant and Grant 1979, and Kleindorfer et al. 2005, Kleindorfer 2007, Kleindorfer and Dudaniec 2016, Kleindorfer et al. 2016 for related work on the island of Floreana), including in the *S. pedunculata* forest at Los Gemelos, where on average nests are found at 5.9 m, 1 m below the top of the canopy (unpublished data provided by A. Cimadom). *Philornis downsi* not only visits bird nests to lay eggs but also may be using bird nests for encountering mates (Pike et al. 2021). In our study, both male and female *P. downsi* were found at low height levels within the forest, which suggests that hill-topping or aerial leks (Wilkinson and Johns 2005, Kokko et al. 2014) are less likely to be reproductive behaviors associated with this fly and support the hypothesis that resource-based mating may be occurring in or around bird nests.

In this study, we saw that the flight layer occupied by *P. downsi* was lower in height than other insects commonly caught in traps, which were caught in the greatest numbers at and above the canopy, between 5.1 and 11.5 m. This may be due to differences in mating behaviors but also resource use. *Scalesia pedunculata* flowers are typically found at the end of branches and project outwards from the top of the canopy (McMullen 1999). If some of the other insects trapped use flowers of *S. pedunculata* as a resource it could help explain their relatively higher distribution in the canopy. *Philornis downsi*, on the other hand, is attracted to and feeds on fermenting fruits, including invasive blackberry (Cha et al. 2016, Fessl et al. 2018, Causton et al. 2019) that are closer to the forest floor. Finer scaled taxonomic identification of a subsample of insects trapped would help to elucidate the extent to which trap rates of different species reflect their diet.

Previous work in a *Scalesia* forest on Floreana Island has shown increased trap rates of *P. downsi* in higher traps, which correlated with elevated nest parasitism rates by *P. downsi* in higher nests (Kleindorfer et al. 2016). Although our results qualitatively contrast with these findings—more flies were caught in lower traps—it is important to take into consideration that the study on Floreana Island deployed traps only within the flight layer occupied by *P. downsi* (2–7 m). The current study, on the other hand, considered a broader range of heights finding that both in and outside of the bird breeding season most *P. downsi* were caught in between 3.1 and 7.5 m. This provides evidence that the distribution of *P. downsi* is height-limited.

Kleindorfer et al (2016) also suggested that higher traps caught more females and that traps at intermediate heights caught more male *P. downsi*. This finding was not replicated in the current study, the sex ratio (0.54) was statistically consistent across all trap heights. The previous study only trapped *P. downsi* in the bird breeding season, whereas the current study considered trap rates across the year, as such, methodological differences likely contribute to the discrepancies in the results; sex ratio data are particularly sensitive to sampling and statistical methods (Brown and Silk 2002).

We saw qualitatively similar seasonal and weather-dependent patterns *for P. downsi* and other insects collected: trap rates of *P. downsi* and other Diptera as well as moths and *P. versicolor* all peaked between April and May (weeks 15–20), lagging behind the start of the hot season in January, which is typically associated with short bursts of heavy rainfall (Trueman and d’Ozouville 2010). These rains lead to rapid growth of vegetation and increasing abundance of plant food resources including flowers and fruits and the insects and birds that feed on them (Grant 1986, Peck 2001, Roque-Albelo 2006). Hot season peaks are common in insect species and relate to increased reproduction when food resources increase in abundance (Kishimoto-Yamada and Itioka 2015, see also Parent et al. 2020 for evidence from *P. versicolor* in Galapagos). All insect species collected responded similarly to weather conditions: trap rates increased with increasing temperature, rainfall, and relative humidity, which aligns with the hot season peaks we observed in the temporal analysis. The significant interaction effects between weather and trap height that we observed were due to stronger effects of temperature, rainfall, and humidity in traps at heights which caught more individuals: traps that caught few individuals did so consistently, regardless of the weather or time of year.

The seasonal and height-dependent variation in trap rates that we observed for *P. downsi*, relative to other commonly caught species, provides new information about the ecology of this invasive species in Galapagos which can be used to design more effective control regimes. Our findings argue against deploying traps above the canopy and suggest setting traps between 3 and 7.5 m (in forests where the canopy layer averages 7–8 m), where *P. downsi* activity is limited to maximize the efficiency of collection and control. In areas with a similar canopy height and story structure to the endemic *S. pedunculata* forest that was studied here, focusing mass trapping below the main canopy, in the main *Philornis* flight layer may render control efforts more efficient and reduce by-catch. These results along with the findings of researchers working on developing lures for trapping (Cha et al 2016, Mieles 2018) will allow us to improve trapping techniques for this invasive fly and help ensure that the unique birdlife of Galapagos is safeguarded.

## Data Availability

Data and code currently archived on github: https://github.com/DrBecky-B/Philornis-trap-height, will be made available on dryad on acceptance.
